# Induction of diabetes in cynomolgus monkey with one shot of analytical grade streptozotocin

**DOI:** 10.1002/ame2.12109

**Published:** 2020-03-20

**Authors:** Zhengzhao Liu, Ying Lu, Wenbao Hu, Hidetaka Hara, Yifan Dai, Zhiming Cai, Lisha Mou

**Affiliations:** ^1^ Shenzhen Xenotransplantation Medical Engineering Research and Development Center Institute of Translational Medicine Shenzhen Second People's Hospital The First Affiliated Hospital of Shenzhen University Health Science Center Shenzhen China; ^2^ Movement System Injury and Repair Research Center Xiangya Hospital of Central South University Changsha China; ^3^ Jiangsu Key Laboratory of Xenotransplantation Nanjing Medical University Nanjing China; ^4^ Xenotransplantation Program/Department of Surgery The University of Alabama at Birmingham Birmingham AL USA

**Keywords:** analytical grade, diabetic model, nonhuman primate, STZ

## Abstract

**Backgrounds:**

Streptozotocin (STZ)‐ induced diabetic monkey is a wide used preclinical animal model for the investigation of diabetes such as islet transplantation and development of diabetic drugs. There are serious side effects of this method, including nausea, emesis, weight loss, liver damage, renal failure, and metabolic acidosis. In order to reduce the side effects, diabetic monkeys were induced using clinical‐grade STZ. However, clinical‐grade STZ is not available in China. Here, we establised a method by using 100 mg/kg analytical‐grade STZ to induce complete diabetes in cynomolgus monkey without generating adverse effects to liver and renal.

**Methods:**

Three cynomolgus monkeys were used in this study. 100 mg/kg STZ dissolved in normal saline and infused through vein line in 5 minutes after indwelling catheter in the carotid artery and jugular vein. After the STZ administration, blood glucose levels were examined every 1 or 2 hours in the first 48 hours. Then, blood glucose levels were examined twice per day during the first week after the STZ injection. Insulin and C‐peptide levels were measured by ELISA. Blood chemistry of hepatic and renal function tests were performed. Insulin and glucagon expression in the islet of diabetic monkey and normal monkey were examined by immunohistochemistry assays.

**Results:**

The stimulated C‐peptide level (Intravenous glucose tolerance test) which is less than 0.5 ng/mL, the triphasic blood glucose response and the destroyed β cell suggested the complete induction of diabetes model. No apparent adverse effects were observed including no signs of vomiting and toxicity after STZ injection.

**Conclusion:**

In summary, we established a safe and reproducible STZ‐induced diabetic cynomolgus monkey model for islet transplantation which will be used to develop novel approaches for the treatment of diabetes.

**Abbreviations**
ALB: albumin
ALT: alanine aminotransferase
BUN: blood urea nitrogen
Cr: creatinine
GGT: r‐glutamyltranspeptidase
GLUT2: glucose transporter 2
IVGTT: intravenous glucose tolerance test
NHP: nonhuman primate
STZ: streptozotocin
T1D: type 1 diabetes
TBA: total bile acid
TP: total protein

## 1 INTRODUCTION

Type 1 diabetes (T1D) is the top two common chronic illness in children.^1^ It is estimated that there are now more than 1 110 100 children and adolesents below 20 years living with T1D.^2^ Animal model of this disorder contribute to treatment of T1D such as evaluating isolated islet grafts and new immunosuppressive drugs for clinical applications.^3^, ^4^ But the lack of a reproducible procedure to generate a diabetic nonhuman primate (NHP) model and the difficulties in maintaining the NHP model hindered the progress of diabetes research. Therefore, a reliable procedure to establish a NHP animal model of T1D would be particularly useful in preclinical trials of novel therapeutic approaches for T1D treatment.

There are several methods of inducing T1D in NHPs, such as complete pancreatectomy, injection of streptozotocin (STZ), etc.^5^, ^6^ Besides, alloxan has been reported to chemically induce a diabetic state in NHPs,^7^ which was reported works selectively on β cell of rodents in recent study.^8^, ^9^ The most utilized model of T1D in mouse is the nonobese diabetic (NOD) mouse that spontaneously develop diseases with similarities to human T1D.^10^, ^11^, ^12^ Additionally, genetically modified animal models of T1D are mainly performed in the rodents, but not NHPs,^13^ such as Ins2Akita mice^14^ and humanized mice with aspects of human immune system.^15^ There are limited transgenic NHP model of human diseases reported such as Huntington's and autism disease monkeys.^16^, ^17^, ^18^ Above all, pancreatectomy has significant drawbacks. The surgery is complicated, and it affects the digestion system due to pancreatic insufficiency. Additional postoperative nursing care is needed as the animal is insulin‐dependent and it shows abnormal digestive system.^1^

An alternative approach to pancreatectomy is the administration of STZ. STZ is a broad‐spectrum antibiotic which may be up‐taken by glucose transporter GLUT2, causing DNA alkylation and destroying the insulin producing β cells.^9^ STZ is a drug used for the treatment of metastatic islet cell carcinoma of the pancreas in clinical. Regarding the toxic side effects, the optimal dose needed to induce a diabetic state is also a challenge for the investigator to produce a permanent diabetic state without adverse effect consistently. In rhesus monkey, 30 mg/kg is not sufficient to induce the monkey diabetic and need higher concentration of more than 45 mg/kg.^19^, ^20^ It is reported that Rhesus macaques (*Macaca mulatto*) received 140 mg/kg of STZ developed T1D and became insulin dependent for more than 1 year without apparent reversal of diabetes.^21^ In another study, Shibata et al test 100, 125, and 150 mg/kg STZ in Rhesus monkey (*Macaca mulatto*) and found that 100 mg/kg of STZ is not sufficient and 150 mg/kg was toxicity, whereas only 125 mg/kg of STZ could reliably induce diabetes without side effects.^22^ Previous studies also reported that 30 mg/kg STZ is successful for inducing diabetes in cynomolgus monkey.^23^ In another study, Koulmanda et al found that 55 mg/kg of STZ was the optimal dosage to induce diabetes in cynomolgus monkeys (*Macaca fascicularis*) and higher dose has more adverse effect, but reports were not successful even with 60 mg/kg STZ.^6^, ^24^ Conversely, larger dose of 150 mg/kg STZ were also used to induce diabetes in cynomolgus monkey with minimal adverse effects and complications by another group.^1^ Rood et al also reported that cynomolgus given 150 mg/kg Zanosar STZ becoming completely diabetic without adverse effects.^6^ Zou et al tested 60, 68, and 100 mg/kg STZ to induce diabetes in cynomolgus monkey and found that 68 and 100 mg/kg of STZ successfully induced a stable diabetic model.^25^ The different dosage for successfully induction of diabetic monkey may due to the differences in the age of the monkey and the effective dose of the drug after dissolving in various solutions such as citrate buffer (PH = 4.5) and normal saline.^3^

As treatment of T1D in NHPs also requires twice a day for blood glucose determination and administration of insulin to maintain blood glucose levels within the range 2.2‐11.1 mmol/L,^26^ the subcutaneous vascular port system provided a safer and easier method for daily access for intravenous sampling and drug delivery.^27^ However, clinical‐grade STZ used in these studies is not available in China.

In this study, we induce diabetes in three cynomolgus monkeys with consistent results, using analytical‐grade STZ. We found that a single dose of 100 mg/kg STZ dissolved in normal saline that were immediately injected into jugular vein through subcutaneous vascular access ports prior putted in animals resulted in consistent, reliable, and safe induction of hyperglycemia. Moreover, diabetic monkeys were treated with 1.2‐3.6 U/d insulin twice daily, without apparent adverse effect and ketoacidosis for at least 2 weeks. We placed subcutaneous vascular access ports in the carotid artery and jugular vein of all animals. This protocol is safe, easy, and reproducible. The diabetic model is useful for preclinical trials for the treatment of T1D in human beings, including the technique of islet cell transplantation.

## 2 MATERIALS AND METHODS

### 2.1 Animals

Three cynomolgus monkeys (3.75‐3.85 kg, female, age 3‐5 years), which were free of Mycobacterium tuberculosis, Shigella, Salmonella, Helminths, Ectoparasites, Entamoebahistolytica, and herpes B virus, were used in this study. Animal care and experiments were conducted at the Landao Bio monkey facility in Guangdong, China, under the guidance of the Animal Laboratory Protocol established by the Guangdong Bureau of Science and Technology and approved by the Institutional Animal Care and Use Committee (IACUC). Monkeys were housed in individual standard cages. The house condition and care of the animal was performed following the previously reported methods.^1^ Room temperature was set at 25°C, and humidity was kept between 40% and 70%. The air was exchanged 12 times hourly. Lights were on from morning to night. Water supply is continuous and commercially monkey food plus fruits were supplied daily twice. The animals were conditioned for a minimum of 1 month prior to the start of the experiment.^1^The work has been reported in accordance with the ARRIVE guidelines (Animals in Research: Reporting In Vivo Experiments).

### 2.2 Induction of diabetes with STZ

Two lines were surgically placed in each monkey.^1^, ^27^ After indwelling catheters in the carotid artery and jugular vein to facilitate drug administration and serial blood sampling, all monkeys were fasted for 12 hour prior to receiving a single (100 mg/kg) intravenous injection of STZ. Giving (0.12 mg/kg, iv) Tropisetro hydrochloride prior to STZ administration to avoid vomiting. 100 mg of STZ (Sigma‐S0130) was first dissolved into 5 mL of saline, and then add to 15 mL cold saline to a final volume of 20 mL. STZ was administered via the jugular vein over a 5‐min period immediately after dissolving.

### 2.3 Insulin treatment of diabetic monkeys

Insulin treatment was performed as previously described.^1^ Briefly, blood glucose levels were monitored twice per day during the first week after the STZ injection using a Glucotrend monitor (Roche Instruments, Basel, Switzerland). Diabetic monkeys were treated with two injections of insulin per day to maintain blood glucose within the range 2.2‐11.1 mmol/L and to prevent metabolic dysfunction. Insulin doses were determined via previously determined scale, which is as follows: glucose levels <11.1 mmol/L = no insulin; glucose levels between 11.1 and 16.6 mmol/L = 1‐2 U of insulin; glucose levels between 16.6 and 22.2 mmol/L = 2‐4 U of insulin; and glucose levels >22.2 mmol/L = 4‐6 U of insulin.^1^

### 2.4 Intravenous glucose tolerance test (IVGTT) and C‐peptide analysis

IVGTT was performed before and after STZ injection. Glucose (0.5 g/kg of body weight) was injected into the femoral vein. Blood glucose levels were recorded at 0, 5, 15, 30, 60, and 90 min with a Glucotrendmonitor. Plasma was also collected at the same time points for insulin (Insulin (IRI) ELISA kit, SIMENS, Germany) and C‐peptide (C‐peptide (CpS) ELISA kit, SIMENS, Germany) measurements. Insulin and C‐peptide levels measured by chemiluminescence machine (ADVIA Centaur®XP, SIMENS, Germany, insulin detection level is 0.34 mU/L).

### 2.5 Blood chemistry

For hepatic and renal function tests, monkey serum was collected from tail vein before and 1 week after STZ administration. The parameters examined include: Total protein (TP), albumin (ALB), total bile acid (TBA), alanine aminotransferase (ALT), r‐glutamyltranspeptidase (GGT), blood urea nitrogen (BUN), and creatinine (Cr).

### 2.6 Histological examination

Normal pancreatic tissue was obtained from a necropsy from cynomolgus monkey. This monkey was used for the experiments not involving diabetes or pancreatic function and was healthy when sacrifice. The diabetic pancreas was obtained from the three monkeys during necropsy after STZ injection. Pancreatic tissue was then fixed in 4% paraformaldehyde (PFA), followed by embedding in paraffin, and cutting into 5‐µm‐thick sections. The sections were de‐paraffin treated, rehydrated, antigen fixed, and stained using an immunohistochemistry technique to visualize β (insulin) and α (glucagon) cells. Briefly, the sections were dewaxed with xylene and ethanol, redydrated with ethanol gradient, blocked with 1% bovine serum albumin at room temperature for 1 hour, and then incubated with primary antibodies overnight at 4℃. The primary antibodies were guinea pig anti‐human insulin (1:100; Cell Signaling Technology) and mouse anti‐human glucagon (1:100; Cell Signaling Technology). The sections were then incubated with donkey anti‐guinea pig IgG or goat anti‐mouse IgG (1:200; Maixin) followed by DAB staining. Images were captured using a Leica2000U microscope (Leica).

### 2.7 Statistical analysis

After STZ administration and immunohistochemistry technique to visualize β (insulin) and α (glucagon) cells, number of β‐ cell or α‐cell per islet area in normal and diabetic monkeys were counted (β‐ or α‐cell number/10^4^ μm^2^) (n = 20, 20 slides in total from three monkeys). Mean values and standard error of the mean (SEM) were calculated using the Prism‐6 software (Graphpad Software).

## 3 RESULTS

### 3.1 High blood glucose level and cessation of C‐peptide production after STZ administration

STZ was infused into the jugular vein, fasting blood glucose level, insulin, and C‐peptide concentrations were measured before and after STZ administration (Table 1). All animals (n = 3, M16001, M16004, M16005) became persistently hyperglycemic. The blood glucose concentrations is >11.1 mmol/L within 36 hours after injection with STZ; the insulin level decreased from 59.33 mU/L on average before STZ to <3.48 mU/L after STZ; the C‐peptide concentration changed from 3.53 ng/mL average before STZ to <0.5 ng/mL after STZ. After STZ injection, all monkeys required 1.2‐3.6 units of additional insulin twice a day to maintain blood glucose. No nausea and vomiting were observed after STZ administration and no signs of toxicity were found to the liver and the renal.

**TABLE 1:** Fasting BG levels, insulin, and C‐peptide values in pre‐STZ and post‐STZ administration

	M16001		M16004		M16005	
	Pre	Post	Pre	Post	Pre	Post
BG levels (mmol/L)	4.4	17.8	4.1	17.2	3.9	12.4
Insulin (mU/L)	119.06	<3.48	10.02	<3.48	48.92	4.53
C‐peptide (ng/mL)	6.69	0.33	1.05	0.09	2.79	0.49

Abbreviation: BG, blood glucose.

**FIGURE 1:**
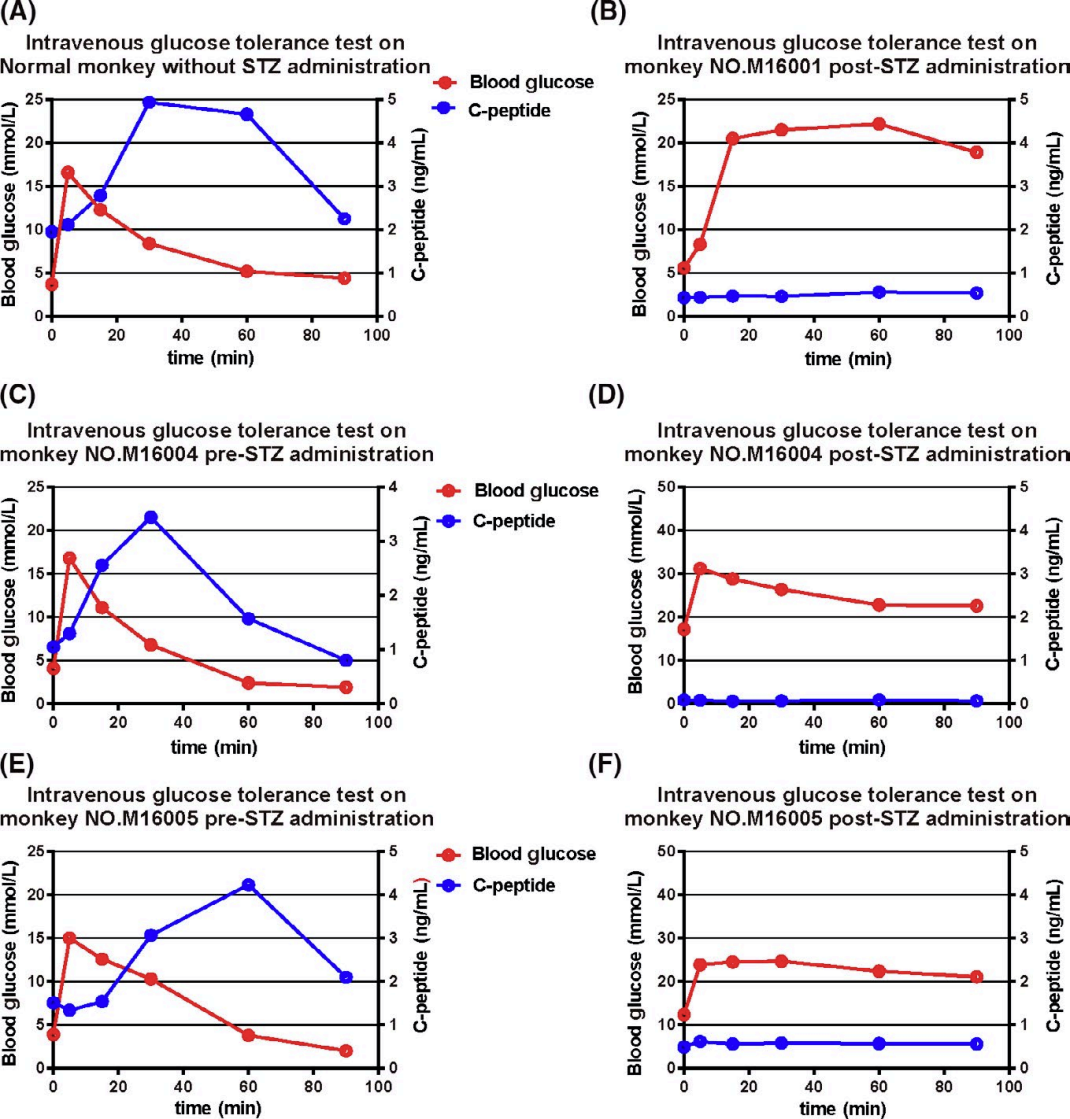
Intravenous glucose tolerance tests Pre‐STZ and Post‐STZ administration. In normal monkeys and in monkeys pre‐administration, increasing blood glucose induced an elevation in c‐peptide in blood. In STZ‐treated monkeys, c‐peptide level is very low even null both before and after glucose injection into blood

Baseline IVGTTs were performed. Fasting blood glucose concentrations recorded before STZ injection was 3.9 ± 0.2 mmol/L. Blood glucose concentrations reached a plateau within 5 minutes after intravenous injection of glucose (16.1 ± 1.0) mmol/L and returned to normal within 60 minutes. Fasting C‐peptide concentrations before STZ treatment was (1.5 ± 0.5) ng/mL. C‐peptide concentrations reached plateau within 30‐60 minutes after intravenous glucose injection (4.2 ± 0.8) ng/mL and returned to basal by 90 minutes (Figure 1A, C, E). IVGTTs were performed again 7 days after injection of STZ. After stopped giving insulin, fasting blood glucose concentrations were increased in all monkeys (11.7 ± 6.0) mmol/L. Blood glucose values reached plateau within 15 minutes after administration of intravenous glucose (26.0 ± 4.6) mmol/L. It kept higher than fasting glucose levels until 90 minutes. Fasting C‐peptide concentrations were decreased in all animals (0.3 ± 0.2) ng/mL. C‐peptide secretions were not stimulated after the administration of intravenous glucose and remained less than 0.5 ng/mL at 90 minutes (Figure 1B, D, F). Insulin levels were under detection level (<3.48 mU/L) after STZ administration (data not shown).

### 3.2 A triphasic blood glucose response to STZ

**FIGURE 2:**
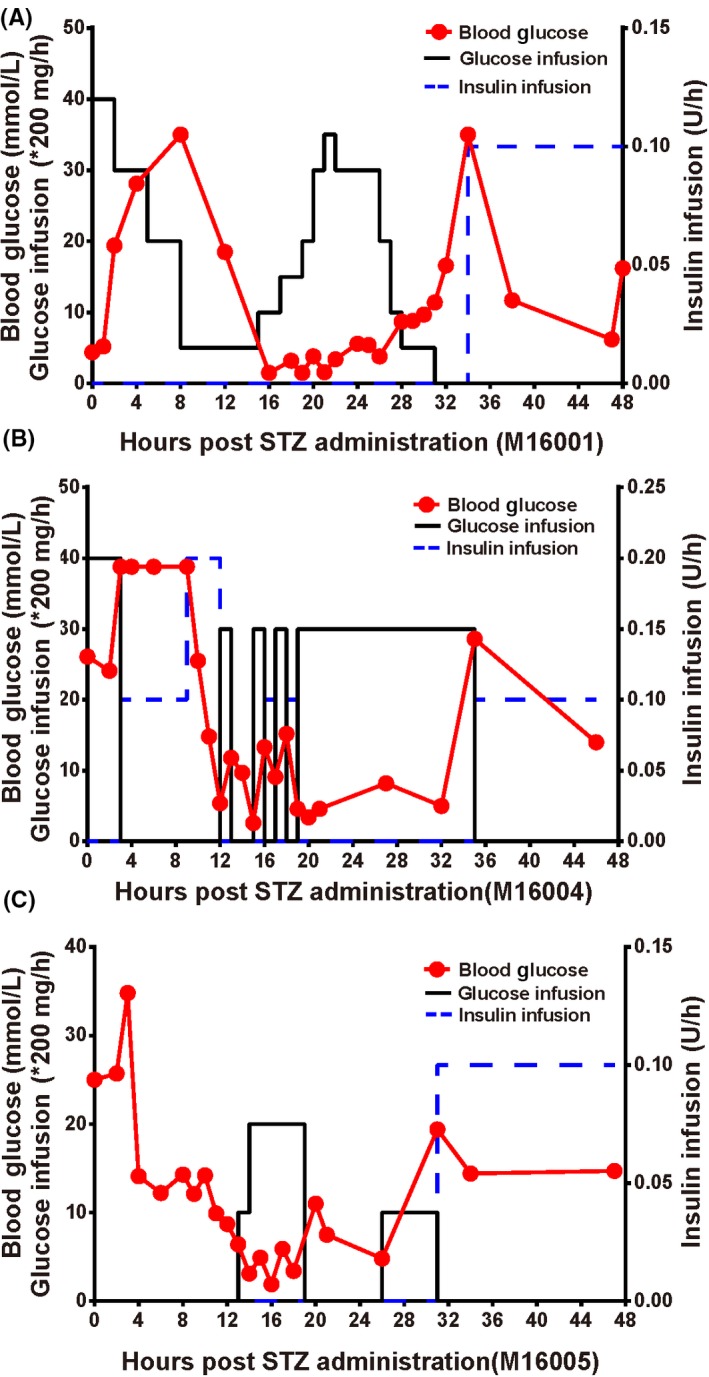
Representative triphasic blood glucose response (red line) after STZ administration in cynomolgus monkeys. Dextrose was administered continuously, trying to keep blood glucose level in normal range (indicated in mg/hr black line). Insulin treatment was started after 30‐34 hours (blue line). M16004 (panel B) was given more insulin at 9 hours after STZ treatment as blood glucose was observed over 40 mmol/L for 5 hours

After the STZ administration, blood glucose levels were examined every 1 or 2 hours in all groups. It is consistent with the previous studies^6^, ^22^ that a triphasic blood glucose curve was recorded in monkeys treated with 100 mg/kg STZ, in responding to STZ treatment (Figure 2). Peak blood glucose appeared after 4‐8 hours post‐STZ; blood glucose decreased steadily until 12‐20 hours, and raised again with persistent hyperglycemia (20‐30 mmol/L) after 32‐36 hours. This triphasic response suggested that the induction of diabetes was complete and successful. Following the STZ injection, all monkeys exhibited hyperglycemia, with fasting blood glucose levels >11.1 mmol/L. Moreover, the IVGTT results suggested dramatic evidence of dampened carbohydrate tolerance in STZ‐treated monkeys (Figure 1). Almost no circulating C‐peptide and insulin could be found after STZ administration and a subcutaneous injection of exogenous insulin was required (1.2‐3.6 U/ d) to avoid metabolic dysfunction.

### 3.3 Histology examination of pancreas, kidney, and liver

**FIGURE 3:**
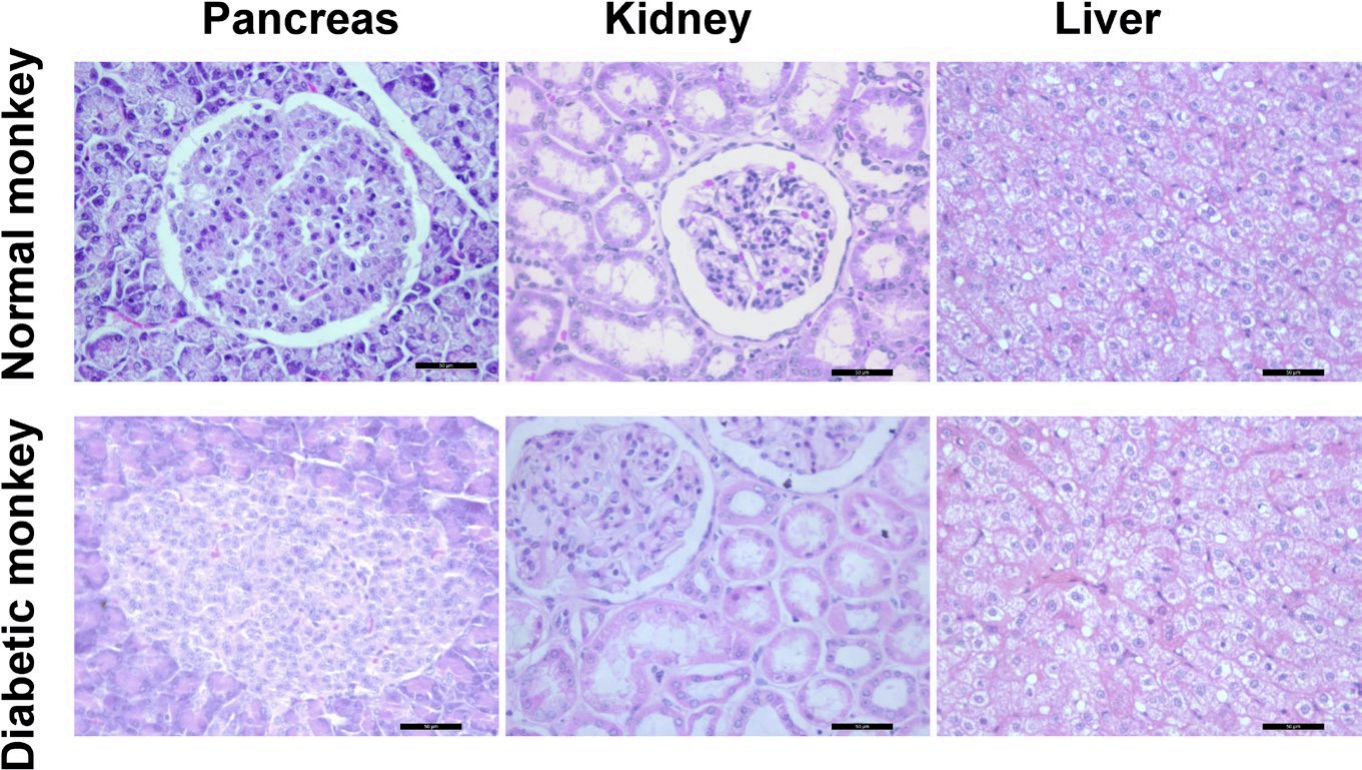
STZ treatment altered pancreas morphology. HE (hematoxylin‐eosin staining) shows the morphology of pancreas, kidney and liver from representative normal and a diabetic monkey. Kidney and liver are normal after STZ treatment. Scale bar, 50 µm

Histology examination of pancreas, kidney, and liver were performed by hematein and eosin stain. Compared with normal monkey, no histopathology abnormalities in liver and kidney were found after STZ administration (Figure 3).

### 3.4 β cells destroyed after STZ administration

**FIGURE 4:**
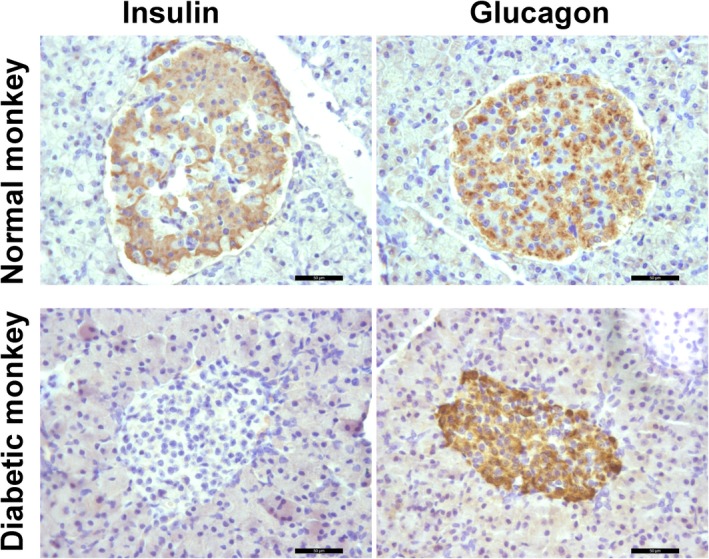
Immunostaining shows insulin and glucagon expression in normal and diabetic monkeys’ islet. Insulin expression vanished indicating β cells were eliminated, whereas glucagon, a market of α cells, expression seemed to be elevated in diabetic monkeys. Scale bar, 50 µm

**FIGURE 5:**
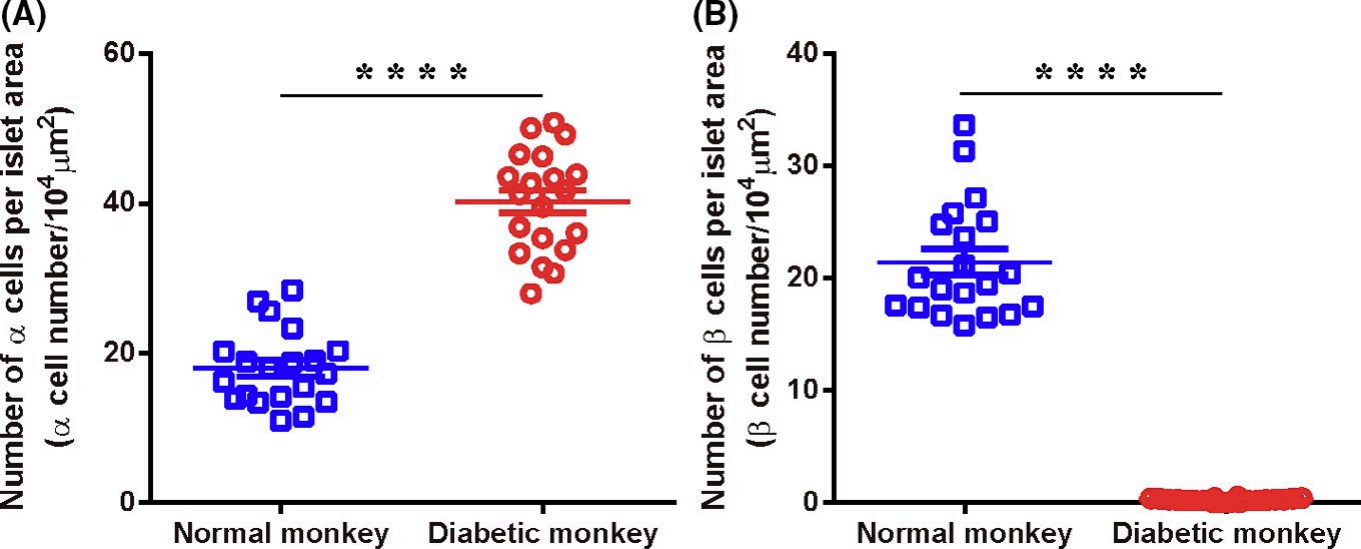
Quantitative of α‐ and β‐cell number in islets with or without STZ treatment. A, Number of β‐cell per islet area in normal and diabetic monkeys (β‐cell number/10^4^ μm^2^). B, Number of α‐cell per islet area in normal and diabetic monkeys (α‐cell number/10^4^ μm^2^). Data are presented in mean ± SEM. n = 20 (20 slides in total from 3 monkeys’ pancreas). ****P < .0001

Immunohistochemistry assays showed that, although glucagon expression was persistent in the residue islets of STZ‐treated monkeys, insulin expression is disappeared (Figure 4). When β‐cell numbers were calculated per islet area, a significant reduction in β‐cell numbers was observed comparing with the control group (Figure 5A). In contrast, the numbers of α‐cell per islet were markedly elevated in the post‐STZ monkeys in comparing with the control group (Figure 5B). This ratio is relative since α cells are the predominant relative population in the islet after β cells are destroyed. These suggested that pathologic changes were limited to β cells in the pancreas without affecting other organs functions (Figures 4 and 5).

### 3.5 No additional signs of toxicity were observed

The blood glucose levels were found dramatically increased on the second day post‐STZ. Table 2 summarized the blood chemistry of hepatic and renal function tests results before and after 1 week of STZ treatment. The results showed that globulin, alanine aminotransferase, and blood urea nitrogen raised slightly in one case but not others. Urine glucose was detected within 72 hours of STZ administration in all the three monkeys. Urine ketones were negative after STZ administration. We did not observed significant gross lesions in any animals at necropsy. Kidney function was also normal for diabetic monkeys after the STZ injection. Other hepatic and renal parameters measured were similar to reference of diabetic monkeys (Table 2).

**TABLE 2:** Hepatic and renal function pre‐STZ and post‐STZ administration^28^

	Reference range	M16001		M16004		M16005	
		Pre	Post	Pre	Post	Pre	Post
TP (g/L)	65‐85	N/A	71.3	77.2	77.5	78	78.9
ALB (g/L)	40‐55	N/A	41.7	46.9	37.4	46.8	39.3
GLB (g/L)	20‐40	N/A	29.6	30.3	40.1	31.2	39.6
TBA (μmol/L)	0‐12	N/A	4.3	2	1.3	27.9	18.5
ALT (U/L)	22‐243	64	105	75	65.0	20	22
GGT (U/L)	10‐60	N/A	40	57	50	55	50
BUN (mmol/L)	1.8‐6.0	3.6	6.1	5.4	5.4	5.4	3.4
Cr (μmol/L)	44‐133	85.8	71.4	67	49.0	72	78
CO_2_ (mmol/L)	20‐30	13	12	30.9	30.3	10.8	15.1

Abbreviations: ALB, albumin; ALT, alanine aminotransferase; BUN, blood urea nitrogen; CO_2_, combining carbon dioxide; Cr, creatinine; GGT, r‐glutamyltranspeptidase; GLB, globulin; N/A, not available; TBA, total bile acid; TP, total protein.

### 3.6 No changes in the body weight after STZ administration

The body weights of all three monkeys were recorded at 1 week and 2 week after STZ injection. Although the body weights decreased in mice model after STZ‐injection in previous studies, the monkeys’ body weights kept stable during the first 2 weeks post‐STZ in our study (Table 3). No significant change in the body weight after STZ administration was observed.

**TABLE 3:** Body weights before and 1 or 2 week post‐STZ administration

	Body weight		
	Pre	Post (1 week)	Post (2 weeks)
M16001	3.75 kg	3.62 kg	3.80 kg
M16004	3.7 kg	3.67 kg	3.56 kg
M16005	3.85 kg	3.77 kg	4.00 kg

## 4 DISCUSSION

Establishing an animal models for diabetes studies, especial setting up an NHP model, are critical for developing novel methods to cure diabetes and preclinical trial for T1D. Several protocols that established diabetic NHP model by STZ administration in cynomolgus and rhesus monkeys.^1^, ^19^, ^20^, ^21^, ^22^, ^23^, ^24^, ^25^ Different species and ages of monkeys affect the efficacy of STZ treatment.^1^, ^24^, ^25^ The optimal dose of STZ used in nonhuman primates is still controversial. It is hard to balance the efficiency to consistently induce diabetic monkeys and the adverse effects caused by the STZ administration. Small doses (55 mg/kg) failed to induce diabetes in cynomolgus monkeys consistently in different groups.^6^, ^24^, ^25^ Large doses caused adverse effects and complications.^1^, ^6^, ^21^, ^22^ The islets may become more sensitive to STZ with age, and this may explain the varying STZ dosages required to induce diabetes in different groups.^1^, ^24^, ^25^ Furthermore, it is justifiable that because of different ambient temperature, the protocol for STZ preparation and infusion time may cause the different results.^29^ Moreover, there is no access for investigators in China to purchase clinical‐grade STZ. Thus, commercially available analytical‐grade STZ was used to induce diabetic monkeys in this study.

After STZ administration, successful induction of diabetes was confirmed by the persistence of fasting hyperglycemia and by the low level of C‐peptide and insulin.^6^ First, diabetes was rendered with fasting blood glucose level >11.1 mmol/L in combination with a stimulated C‐peptide <0.5 ng/mL. Second, complete and successful induction of diabetes should have the appearance of triphasic blood glucose response after STZ administration and disappearance of β cells.^6^, ^30^, ^31^ The dose of STZ positive correlated with the development of renal and hepatic damage.^24^, ^25^ This is shown by an elevation in BUN, serum creatinine, and hepatic enzymes.^1^, ^25^ Therefore, it is essential to find the optimal dose of STZ to induce diabetes reliably and without apparent adverse effects.

In this study, we successfully induced diabetes in cynomolgus monkeys without β cell remaining and without generating adverse effects to liver and renal using 100 mg/kg STZ. In this study, blood glucose, plasma insulin and C‐peptide, and hepatic and renal function which were associated with a diabetic state were recorded before and after STZ administration in these animals. Within 36 hours after STZ treatment, a triphasic response in blood glucose appeared. This has been documented in rodents^30^, ^31^ and monkeys.^6^, ^32^ Initial reduction in insulin may be due to immune reactions including inflammation and reduced ability of β cells to secret insulin,^9^ which explained the phase I increase in blood glucose level. Further disruption of the β cells by STZ leads to an extensive release of insulin resulting in phase II reduction in blood glucose level. Finally, stable hyperglycemia in phase III is due to the complete loss of β cells.^6^ This optimal dosage of STZ provides an useful procedure to set up NHP diabetic model for islet transplantation. Blood glucose reaches to plateau after 4‐8 hours (compared with 2‐7 hours in rat), followed by a steady decrease until 12‐20 hours (compared with 8‐12 hours in rat), and a subsequent persistent high blood glucose level (20‐30 mmol/L) after 36 hours (24 hour in rat). This triphasic response indicated the complete and successful induction of diabetes. Pancreatic islet immunohistochemistry results suggested a nearly complete loss of β cells in the islets of all monkeys treated with STZ. This was consistent with the blood glucose levels, which were persistently high. The side effects of STZ mainly include hepatic adrenal injury. Although we observed a slight change in the indexes of hepatic and renal function (ie, ALT, TBA, and BUN) within 1 week post‐STZ, the numerical values were still in the normal range.

In conclusion, diabetic cynomolgus monkeys can be reliably established at the dose of 100 mg/kg using analytical‐grade STZ without significant hepatic or renal impairment. This may be valuable for the future research of diabetes model for NHP, especially for those who are not able to access clinical‐grade STZ.

## CONFLICT OF INTEREST

None.

## AUTHOR CONTRIBUTIONS

ZL, WH, YL, YD, ZC, and LM conceived the study and LM directed the study. ZL, WH, and YL performed and analyzed most of the work. HH supported the work technically and supervised the work. ZL and YL recorded the glucose level and perform the insulin injection. ZL, WH, and YL performed and analyzed the insulin and glucagon staining. And ZL wrote the paper with substantial input from WH and YL.
